# Effects of metformin on blood and urine pro-inflammatory mediators in patients with type 2 diabetes

**DOI:** 10.1186/s12950-016-0142-3

**Published:** 2016-11-24

**Authors:** Wei Chen, Xiaojie Liu, Shandong Ye

**Affiliations:** 1 School of Medicine, Shandong University, Jinan, Shandong China; 2 Department of Nephrology, Affiliated Anhui Provincial Hospital, Anhui Medical University, Hefei, Anhui China; 3 Department of Cadre Wards, Affiliated Anhui Provincial Hospital, Anhui Medical University, Hefei, Anhui China; 4 Department of Endocrinology, Affiliated Anhui Provincial Hospital, Anhui Medical University, Hefei, Anhui 230001 People’s Republic of China

**Keywords:** Metformin, Diabetes, Inflammatory responses, Hypertension, Renal function

## Abstract

**Background:**

Metformin has been used for the treatment of type 2 diabetes by suppressing hepatic gluconeogenesis. It has been shown that the subclinical inflammatory responses play important roles in the pathogenesis of type 2 diabetes. In the present study, we determined the effects of metformin on the levels of pro-inflammatory cytokines (i.e., IL-6, TNF-α, and MCP-1) and anti-inflammatory mediator IL-10 in blood and urine of patients with type 2 diabetes. There were 210 patients with type 2 diabetes, which were randomized into metformin (*n* = 112) and non-metformin (gliclazide, acarbose, and repaglinide, *n* = 98) groups. The levels of cytokines were measured by the ELISA.

**Results:**

We found that metformin reduced the levels of IL-6 in blood and MCP-1 in urine, but increased IL-10 levels in blood of patients with type 2 diabetes. There were no significant differences of TNF-α between metformin and non-metformin groups. Furthermore, compared to individual drug treatment, metformin significantly reduced the levels of serum IL-6 and TNF-α, as well as urine MCP-1. When the patients were stratified based on the durations and doses of metformin, we found that there was only change (i.e., increase) in serum IL-10 levels in patients with metformin for more than 1 year compared to treatment for less than 1 year. Metformin (1.5 g) treatment reduced the urinary levels of MCP-1 as compared with dose of 1.0 g in patients with type 2 diabetes.

**Conclusion:**

Metformin reduces inflammatory responses without influence on renal function in type 2 diabetic patients.

## Background

Diabetes mellitus is a metabolic disease, which is characterized by the hyperglycemia due to either insulin insufficiency or resistance. There are more than 90% cases with type 2 diabetes where the cells in the body show reduced reaction to insulin (insulin resistance). Diabetes is the seventh most common cause of death in the United States, and it causes a variety of health complications including heart disease, blindness, kidney failure, and lower-extremity amputations. Numerous pathogenic processes are implicated in the development of diabetes, which result from the β-cell destruction in the pancreas leading to insulin deficiency or from the abnormalities that cause insulin resistance in target tissues [1, 2]. The abnormal inflammatory responses have been shown to play important roles in the pathogenesis and progression of type 2 diabetes [3–5]. For instance, pro-inflammatory cytokines including interleukin (IL)-6 and tumor necrosis factor (TNF)-α enhance insulin resistance, and are associated with increased risk of type 2 diabetes [6–9]. In contrast, IL-10, an anti-inflammatory cytokine, is reduced in patients with type 2 diabetes [10]. Therefore, the therapeutic treatments with anti-inflammatory properties would be beneficial to the management of type 2 diabetes.

Metformin is the first-line therapy for the treatment of type 2 diabetes by repressing the hepatic gluconeogenesis [11–13]. The molecular mechanism of metformin is associated with the activation of AMP-activated protein kinase (AMPK) and protein kinase A (PKA) as well as the inhibition of the mitochondrial respiratory chain (complex I) and glycerophosphate dehydrogenase. It has been suggested that metformin improves metabolic parameters such as hyperglycemia, insulin resistance and atherogenic dyslipidemia, thereby reducing chronic inflammatory responses [14, 15]. However, it is not clear whether metformin has any effects on inflammatory responses in the systemic circulation and urine of patients with type 2 diabetes. In the present study, we determined the effects of metformin with different doses and durations on the levels of pro-inflammatory cytokines (i.e., IL-6, TNF-α, and MCP-1) and anti-inflammatory mediator IL-10 in blood and urine of patients with type 2 diabetes.

## Methods

### Patient enrollment

There were 210 patients diagnosed with type 2 diabetes during the period of January 2015 to December 2015 in the Anhui Provincial Hospital. The characteristics of these patients were shown in Table 1. The criteria for patient inclusion were shown as the following: 1) All patients are diagnosed according to the WHO diagnostic guidelines (1999 version) for type 2 diabetes; 2) ages are between 42 and 70; 3) 4.8% ≤ glycated hemoglobin A1c ≤ 7.5%. The criteria for patient exclusion were described as the following: 1) Patients with acute infection in past two months; 2) Any systemic immune disorders; 3) Chronic infectious diseases and tumors; 4) Surgical operation within last three months; 5) Severe hepatic and kidney dysfunction; and 6) Extremely high blood pressure.

**Table 1:** Changes in blood pressure, hepatic and renal biochemistry between metformin and non-metformin groups

Variables	Metformin		Non-metformin		*P* value
	Mean ± SD	N	Mean ± SD	N	
Blood glucose (mmol/L)	6.41 ± 0.98	112	6.33 ± 0.90	169	0.4993
Systolic blood pressure (mmHg)	133.29 ± 11.24	112	134.63 ± 9.43	169	0.2718
Diastolic blood pressure (mmHg)	73.69 ± 7.23	112	74.22 ± 6.78	169	0.5271
Hemoglobin A1c (%)	6.40 ± 0.57	112	6.29 ± 0.60	169	0.1260
Body mass index	25.66 ± 3.64	112	25.18 ± 3.43	169	0.2584
Total cholesterol (mmol/L)	4.71 ± 1.26	112	4.61 ± 0.90	169	0.4265
Triglyceride (mmol/L)	1.90 ± 1.27	112	1.58 ± 0.99	169	0.0205
HDL(mmol/L)	1.13 ± 0.30	87	1.16 ± 0.32	59	0.5051
LDL (mmol/L)	2.66 ± 0.82	112	2.76 ± 0.81	169	0.3443
VLDL (mmol/L)	0.81 ± 0.41	87	0.69 ± 0.45	59	0.0988
Creatinine (μmol/L)	83.02 ± 17.49	112	85.89 ± 32.90	169	0.3979

### Metformin and other anti-diabetic medications

All these 210 patients with type 2 diabetes were randomly divided into metformin (*n* = 112) and non-metformin (*n* = 98) groups. For the metformin group, patients received metformin (1000 mg or 1500 mg) daily through oral administration. For the non-metformin group, patients received one of the following drugs: gliclazide (a sulfonylurea, *n* = 44), acarbose (a glucosidase inhibitor, *n* = 18), and repaglinide (stimulating the release of insulin, *n* = 36) for 12 months.

### Measurement of cytokine by ELISA

Serum was obtained from patient venous blood through centrifugation for 10 min at 1500 rpm. Similarly, the urine was collected followed by the centrifugation, and kept at −80 °C until analysis. The levels of IL-6, IL-10 and TNF-α in serum and monocyte chemoattractant protein-1 (MCP-1) in urine were measured using the corresponding ELISA kits from R&D Systems (Minneapolis, MN, USA) according the manufacturer’s protocols.

### Statistics

All statistical analysis was performed by the Graphpad Prism software, and the differences between two groups (e.g., metformin vs every treatment) were compared using a Dunnett’s post-hoc test following an ANOVA.

## Results

### Biochemical characteristics between metformin and non-metformin groups

As shown in Table 1, there was no significant difference in biochemical parameters including fasting blood glucose, glycated hemoglobin A1c, total cholesterol, or triglyceride between metformin and non-metformin (i.e., gliclazide, acarbose, or repaglinide) treated type 2 diabetic patients.

### Changes in IL-6, IL-10, and TNF-α in serum between metformin and non-metformin groups

**Fig. 1:**
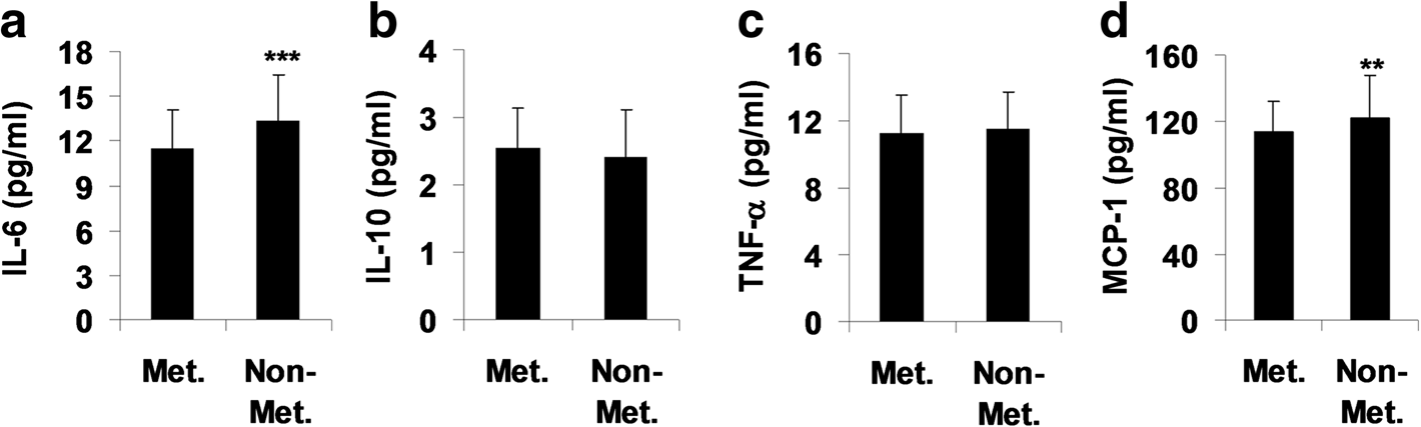
Effect of metformin on cytokines in serum and urine of patients with type 2 diabetes. Serum and urine were collected from 112 metformin (Met.)-treated and 169 non-metformin-treated type 2 diabetic patients for cytokine measurement (**a**, IL-6; **b**, IL-10; **c**, TNF-α; **d**, MCP-1) by ELISA. Data were shown as Mean ± SD. ^**^ *P* < 0.01, ^***^ *P* < 0.001, vs metformin group

To determine any differences in cytokines in serum from patients treated with metformin and non-metformin treated type 2 diabetic patients, we measured the levels of IL-6, TNF-α, and IL-10 (Fig. 1a-c). We found that there were no significant differences in levels of TNF-α or IL-10 in serum between metformin and non-metformin treated type 2 diabetic patients. However, the levels of IL-6 were significant reduced in serum of metformin-treated patients as compared to non-metformin treated patients. These results suggest that metformin has a specific effect on pro-inflammatory mediators in type 2 diabetic patients.

### Changes in MCP-1 in urine between metformin and non-metformin groups

Urinary cytokine might be useful for the early diagnosis and management of patients with diabetic nephropathy [16, 17]. Hence, we measured the MCP-1 levels in urine of patients treated with metformin and non-metformin (i.e., gliclazide, acarbose, or repaglinide). We found that the levels of MCP-1 were significantly reduced in urine of metformin-treated patients as compared to non-metformin group (Fig. 1d). These results indicate that metformin has an inhibitory influence on urinary cytokine in type 2 diabetic patients.

### Comparison of metformin with other individual drugs on cytokines

To further determine the effects of metformin on cytokines as compared to individual drug, we analyzed the levels of cytokines in serum and urine of patients treated with metformin, gliclazide, acarbose, and repaglinide. As shown in Table 2, the levels of IL-6 and TNF-α in serum were significantly reduced by metformin as compared to acarbose and repaglinide, whereas there are no changes in serum IL-10 among these treatments. Compared to gliclazide and repaglinide, metformin treatment significantly reduced MCP-1 levels in urine. Altogether, metformin reduces the levels of inflammatory cytokines as compared to individual gliclazide, acarbose, and repaglinide treatment.

**Table 2:** Comparison of metformin with other drugs on cytokines

Treatment	N	Serum (pg/ml)			Urine (pm/ml)
		IL-6	IL-10	TNF-α	MCP-1
Metformin	54	11.27 ± 2.56	2.50 ± 0.59	11.26 ± 2.14	111.28 ± 21.06
Gliclazide	44	12.24 ± 2.42	2.55 ± 0.69	10.89 ± 2.26	126.42 ± 28.85**
Acarbose	18	12.99 ± 2.73*	2.19 ± 0.74	12.62 ± 3.17*	114.24 ± 22.86
Repaglinide	36	13.02 ± 2.78**	2.56 ± 0.62	11.36 ± 1.98	123.46 ± 14.30**

*P < 0.05

**P < 0.01, vs metformin

### Effects of metformin with different durations on cytokines

**Fig. 2:**
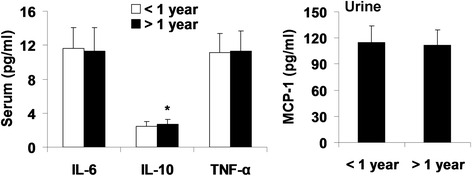
Effect of metformin with different durations on cytokines in serum and urine of patients with type 2 diabetes. Serum and urine were collected from type 2 diabetic patients treated with metformin for less (*n* = 71) and more than 1 year (*n* = 41) for cytokine measurement by ELISA. Data were shown as Mean ± SD. ^**^ *P* < 0.05, vs metformin (<1 year) group

To determine the durations of metformin on cytokines in serum and urine, we divided the type 2 diabetic patients into two groups (<1-year and > 1-year of metformin treatment). No changes in biochemistry were observed between these two groups of patients (Table 3). There was no effect of serum IL-6, TNF-α, or urinary MCP-1 levels between <1-year and >1 year treatments with metformin in type 2 diabetic patients (Fig. 2). The levels of IL-10 were increased in patients treated with metformin for more than 1 year compared to less than 1 year (Fig. 2). These data indicate that metformin has an anti-inflammatory property in a duration-dependent manner.

**Table 3:** Effect of metformin with different durations on blood biochemistry

Variables	Duration (<1 year)		Duration (>1 year)		*P* value
	Mean ± SD	N	Mean ± SD	N	
Blood glucose (mmol/L)	6.45 ± 1.00	71	6.35 ± 0.95	41	0.6092
Systolic blood pressure (mmHg)	132.21 ± 9.76	71	135.17 ± 13.35	41	0.1807
Diastolic blood pressure (mmHg)	73.56 ± 6.89	71	73.90 ± 7.86	41	0.8122
Hemoglobin A1c (%)	6.39 ± 0.56	71	6.41 ± 0.59	41	0.8255
Body mass index	25.51 ± 3.04	71	25.93 ± 4.53	41	0.5601
Total cholesterol (mmol/L)	4.81 ± 1.44	71	4.55 ± 0.87	41	0.3038
Triglyceride (mmol/L)	1.12 ± 0.34	71	2.09 ± 1.49	41	0.0400
HDL (mmol/L)	1.12 ± 0.34	36	1.15 ± 0.23	23	0.7767
LDL (mmol/L)	2.65 ± 0.83	71	2.69 ± 0.81	41	0.7920
VLDL (mmol/L)	0.83 ± 0.39	36	0.77 ± 0.44	23	0.5596
Creatinine (μmol/L)	80.75 ± 15.11	71	86.95 ± 20.59	41	0.0703

### Effects of metformin with different doses on cytokines

**Fig. 3:**
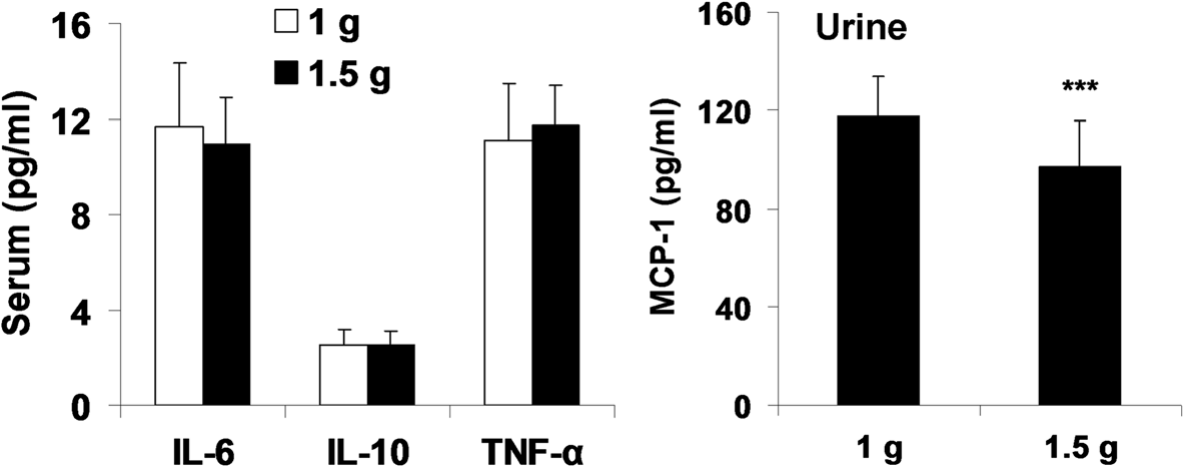
Effect of metformin with different doses on cytokines in serum and urine of patients with type 2 diabetes. Serum and urine were collected from type 2 diabetic patients treated with metformin with 1 g (*n* = 91) and 1.5 g (*n* = 21) for cytokine measurement by ELISA. Data were shown as Mean ± SD. ^***^ *P* < 0.001, vs metformin (1 g) group

To determine the doses of metformin on cytokines in serum and urine, we divided the type 2 diabetic patients into two groups (1000 and 1500 mg of metformin treatment). No significant changes in biochemistry were observed between these two groups of patients (Table 4). We found that there were no effects of serum IL-6, IL-10, or TNF-α between 1000 and 1500 mg treatments with metformin in type 2 diabetic patients (Fig. 3). However, urinary MCP-1 levels were significantly reduced in patients treated with metformin at 1500 mg as compared to 1000 mg of metformin treatment (Fig. 3). These results indicate that metformin reduces inflammatory responses in a dose-dependent manner.

**Table 4:** Effect of metformin with different doses on blood biochemistry

Variables	Metformin (1000 mg)		Metformin (1500 mg)		*P* value
	Mean ± SD	N	Mean ± SD	N	
Blood glucose (mmol/L)	6.44 ± 0.96	91	6.27 ± 1.04	21	0.4684
Systolic blood pressure (mmHg)	133.67 ± 11.16	91	131.67 ± 11.70	21	0.4640
Diastolic blood pressure (mmHg)	73.10 ± 6.73	91	76.24 ± 8.81	21	0.0727
Hemoglobin A1c (%)	6.36 ± 0.57	91	6.55 ± 0.58	21	0.1797
Body mass index	25.64 ± 3.52	91	25.75 ± 4.23	21	0.8989
Total cholesterol (mmol/L)	4.68 ± 1.36	91	4.85 ± 0.76	21	0.5970
Triglyceride (mmol/L)	2.00 ± 1.38	91	1.48 ± 0.45	21	0.0940
HDL(mmol/L)	1.13 ± 0.31	46	1.15 ± 0.30	13	0.7942
LDL (mmol/L)	2.58 ± 0.83	91	3.03 ± 0.66	21	0.0212
VLDL (mmol/L)	0.82 ± 0.42	46	0.75 ± 0.38	13	0.5954
Creatinine (μmol/L)	82.07 ± 15.98	91	87.14 ± 22.93	21	0.2321

## Discussion

In the present study, we found that metformin treatment has specific effects on cytokines (serum IL-6 and urinary MCP-1) as compared to the non-metformin (i.e., gliclazide, acarbose, or repaglinide) treatment type 2 diabetic patients. When compared to individual gliclazide, acarbose, or repaglinide treatment, metformin significantly reduced the pro-inflammatory cytokines in serum and urine. In addition, metformin reduced inflammatory responses in a duration- and dose-dependent manner in patients with type 2 diabetes. These findings suggest that metformin (1000 mg, q.d.) for 1 year has beneficial effects to reduce inflammatory responses in systemic circulation and urine in type 2 diabetic patients.

In addition to diet control and exercise, type 2 diabetic patients commonly require diabetes medications and insulin. The most commonly prescribed medication for type 2 diabetes is metformin with less side-effect of hypoglycemia. As compared to gliclazide, acarbose, or repaglinide, there were no significant changes in biochemical parameters for hepatic and renal function. These results suggest that the medications including metformin for type 2 diabetic patients in this study are safe.

A variety of mechanisms contribute to defective insulin secretion and responses in type 2 diabetes, which including glucotoxicity, lipotoxicity, oxidative stress, and the formation of amyloid deposits in the islets [18, 19]. Interestingly, all of these mechanisms are associated with inflammatory responses [20]. This is corroborated by the findings that chronic inflammatory responses play important roles in the pathogenesis of type 2 diabetes by causing islet dysfunction and insulin resistance in both inflammasome-dependent or -independent manners [21]. Therefore, the ideal anti-diabetic drugs would be possessing anti-inflammatory properties in addition to reducing glucose in blood. In comparison with gliclazide, acarbose, and repaglinide, metformin reduced the levels of serum IL-6, TNF-α, and urinary MCP-1 in patients with type 2 diabetes. In addition, metformin exhibits a specific effect on inflammatory responses at the different doses and durations. Altogether, these findings suggest that metformin exhibits anti-diabetic effects by reducing both glucose levels and inflammatory responses.

It is well-known that metformin treatment in diabetic patients with chronic kidney diseases would be cautious, as it causes lactic acidosis in the setting of renal dysfunction [22, 23]. In our study, we did not find any abnormalities of renal function in these diabetic patients treated with metformin or non-metformin. Future studies are required to determine how metformin reduces MCP-1 reduction in urine without changes in renal function. In addition, the disadvantage of this study is unable to determine the effects of metformin or other anti-diabetic drugs on inflammatory responses through a longitudinal manner.

## Conclusion

Metformin reduces inflammatory responses in addition to glucose reduction in type 2 diabetic patients. There are specific effects on cytokines (serum IL-10 and urinary MCP-1) by the different durations and doses of metformin in patients with type 2 diabetes. Therefore, metformin reduces inflammatory responses in systemic circulation and urine, which contributes to its beneficial effects on type 2 diabetes.

## Abbreviations

AMPK: AMP-activated protein kinase
IL: Interleukin
MCP-1: Monocyte chemoattractant protein-1
PKA: Protein kinase A
TNF: Tumor necrosis factor
